# Resilience and Self-Compassion: Shields Against Age-Related Declines in Oral Health and Shame

**DOI:** 10.7759/cureus.66565

**Published:** 2024-08-10

**Authors:** Christos Tsironis, Fotios Tatsis, Zoe Konstanti, Manolis Mentis, Konstantinos Stolakis, Vasiliki Kotsia, Stefanos Mantzoukas, Elena Dragioti, Mary Gouva

**Affiliations:** 1 Research Laboratory Psychology of Patients, Families and Health Professionals, Department of Nursing, School of Health Sciences, University of Ioannina, Ioannina, GRC; 2 Faculty of Medicine, School of Health Sciences, University of Ioannina, Ioannina, GRC; 3 Department of Educational Sciences and Social Work, University of Patras, Patra, GRC; 4 Research Laboratory Integrated Care, Health and Well-Being, Department of Nursing, School of Health Sciences, University of Ioannina, Ioannina, GRC

**Keywords:** oral, self-compassion, resilience, shame, oral health status

## Abstract

Objective

This study aimed to investigate the complex relationships between demographic factors, oral health indicators, self-compassion, and psychological well-being among elderly individuals by using a path model analysis.

Methods

The findings of this cross-sectional study are derived from data collected from a sample of 204 patients, including 120 females and 84 males, aged between 60 and 92 years, with an average age of 74.2 years (SD = 7.1). Participants took part in assessments including the Geriatric Oral Health Assessment Index (GOHAI) to measure oral health (physical function, psychosocial function, pain/discomfort), self-compassion scale, and measures of shame and "other as shamer" experiences. Demographic information including age and gender was also collected. Path analysis was employed to examine the direct and indirect effects of demographic variables, oral health indicators, and self-compassion on psychological well-being.

Results

Older age was consistently associated with poorer oral health outcomes across all GOHAI oral health subscales. However, resilience and self-compassion appear to exert a correspondingly large positive influence, neutralizing the negative effects of increasing age on physical and psychosocial aspects of oral health-related quality of life. Additionally, resilience was positively associated with better physical and psychosocial function related to oral health while oral health was confirmed to be related to internal and external (social) shame. However, resilience and self-compassion outperformed oral health as far as their effect on internal and external (social) shame is concerned. Gender had minimal effects on most outcome variables.

Conclusions

The findings underscore the importance of addressing both physical and psychological aspects of health in elderly care and oral health interventions. By promoting psychological resilience and self-compassion, healthcare providers can potentially enhance oral health-related quality of life and overall well-being among elderly populations. Our results also highlight that promoting social and leisure activities may be a means of improving mental well-being, enhancing oral health outcomes, and reducing shame-related distress among elderly individuals.

## Introduction

The rapid aging of populations worldwide brings forth significant challenges and opportunities in healthcare [1]. To gain a better understanding of the factors affecting healthcare outcomes, the notion of psychological well-being, characterized by traits such as self-compassion and resiliency, has garnered increasing attention in terms of health outcomes across the lifespan [2-8]. In this context, resiliency corresponds to the ability to adapt and bounce back from adversity [9,10], while self-compassion is seen as a self-attitude that involves treating oneself with warmth and understanding in difficult times and recognizing that making mistakes is part of being human [6,11,12]. Age plays a significant role in shaping self-compassion, with older individuals potentially facing greater challenges and adversities that impact their self-compassion levels [13,14]. An analogous status has been described for resiliency, with older individuals potentially exhibiting varying levels of resilience in response to challenges associated with aging [15-17]. Similarly, gender differences in these traits may exist due to sociocultural norms and experiences unique to each gender [18-20].

Oral health is considered a vital component of overall well-being for the elderly, influencing various aspects of daily life, including eating, communication, and social interaction [21-25]. In research endeavors seeking a more comprehensive understanding of the interplay between oral and mental well-being, shame is frequently cited as a factor leading to psychological distress [26,27]. Shame is a profound and highly self-conscious emotion that exerts a profound influence on an individual's sense of self, overall well-being, and susceptibility to various forms of psychopathology [28]. This emotional response has a broad-reaching impact across multiple domains of mental health, including depression [29-31], anxiety [32], paranoia [33], post-traumatic stress disorder [34], eating disorders [35,36], and personality disorders [37]. This distress, in turn, has adverse effects, such as fostering fear and avoidance behaviors related to seeking dental care [38]. These consequences are even more recognized among older individuals, in whom declining oral health and tooth loss often lead to limited social interaction and diminished self-esteem [39-41].

Both resiliency and self-compassion are recognized as crucial aspects of psychological well-being that may influence oral health outcomes among elderly individuals [42]. Accordingly, the association between these traits and oral health-related quality of life has also been analyzed in the literature [43]. Yet, the specific roles of self-compassion and resiliency in shaping oral health outcomes and their relative effect on oral health and shame-associated feelings have not been adequately explored.

The relationship between oral health and psychopathology has been studied in a sample of older individuals and the significant interplay between oral health, shame, and psychopathology was validated [44]. Specifically, shame was identified as a significant mediator in the relationship between oral health and psychopathology, highlighting the complex pathways through which oral health status may impact psychological well-being in later life. Building upon these insights, this study seeks to further elucidate the mechanisms underlying these relationships and expand our understanding of the role of psychosocial factors in shaping individuals' experiences of oral health-related quality of life and psychological distress.

Specifically, by examining additional psychosocial constructs such as resiliency and self-compassion, a more comprehensive framework for exploring the intricate connections between oral health, shame, and psychopathology among elder individuals is highlighted, shedding light on potential targets for intervention and support services for promoting holistic well-being in aging individuals [45]. Addressing these knowledge gaps is critical for developing targeted interventions and support services tailored to the unique needs of elderly persons [46-49].

## Materials and methods

**Figure 1:**
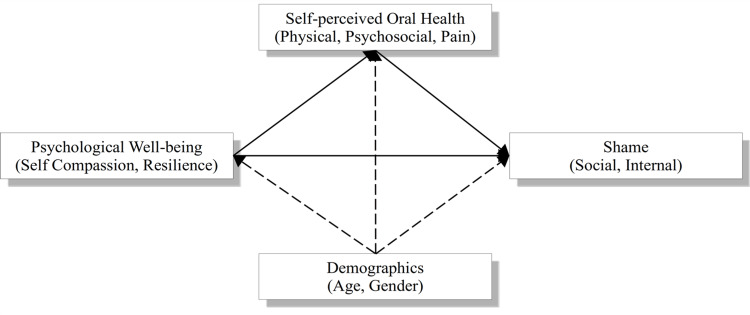
The conceptual model

The objective of this study was to assess the associations between demographic factors (age, gender), psychological constructs (self-compassion, resilience), oral health-related quality of life (physical function, psychosocial function, pain/discomfort), and shame, in the context of a mediation path model. Specifically, the mediation model illustrated in Figure 1 was adopted, wherein oral health serves as a mediating factor in the connection between psychological well-being and shame controlling for the demographic variables of age and gender.

The model aims to address several key questions through path analysis. These include: (1) understanding the influence of demographic factors, such as age and gender, on oral health; (2) examining the role of psychological constructs, specifically self-compassion and resilience, in determining oral health outcomes; (3) exploring the mediating effects of psychological factors between demographics and oral health; and (4) investigating the association between oral health and psychological well-being, particularly the experience of shame. The path analysis framework allows for the examination of direct and indirect relationships among these variables, offering a comprehensive view of how these factors interrelate. This model extends the investigation presented by Tsironis et al. (2024) by incorporating additional psychosocial constructs and examining their interrelationships more deeply [44]. By doing so, the study aims to provide a nuanced understanding of the complex interplay between oral health, psychosocial factors, and overall mental health in aging individuals. This refined model builds upon the foundation laid by previous research, allowing for a more thorough exploration of the underlying mechanisms at play.

Study design and participants

This cross-sectional study was conducted in the Psychology Research Lab for Patients, Families, and Health Professionals at the University of Ioannina in 2023. The cohort consisted of individuals who were currently under the medical care of the primary researcher, without any recognized diagnosis of psychiatric illness, and who had scheduled routine dental appointments. The study's participant pool comprised a total of 204 patients (121 females and 83 males) with ages spanning from 60 to 92 years, (females: 74.3 ± 7.2; males: 74.1 ± 7.0 years) and an average age of 74.2 years (SD = 7.1). Out of the total 204 patients, four males were identified with at least one missing value in the required demographic or psychometric characteristics of the path model, leading to their exclusion from path analysis.

The majority of participants (n = 129, 64.5%) resided in small villages. Additionally, 130 participants (65%) had completed their education up to the primary school level. Furthermore, a substantial proportion of the participants were married (110 participants, 55%), with the majority of them being pensioners (186 participants, 93%). In terms of living arrangements, the study observed that a significant portion of the participants lived either alone (72 participants, 36%) or with a partner (104 participants, 52%).

The survey questionnaire was personally administered by the primary researcher. This method was selected to address potential concerns related to reports suggesting that individuals with lower levels of education might encounter challenges in understanding the intended direction of the answers, as indicated in previous studies [50].

Measurements

Participants completed a series of self-report measures assessing various domains of interest. These measures included the Geriatric Oral Health Assessment Index (GOHAI), the Self-Compassion Scale (SCS), the Resilience Scale (CDRISC), the Experience of Shame Scale (ESS), and the Other As Shamer Scale (OAS).

The GOHAI was used to assess oral health-related quality of life across three domains: physical function, psychosocial function, and pain/discomfort [51]. The GOHAI has demonstrated good psychometric properties, including high internal consistency, adequate test-retest reliability, and construct validity. Participants completed the Self-Compassion Scale (SCS) to measure levels of self-compassion across six dimensions, including self-kindness, common humanity, and mindfulness [6]. The SCS has been validated with strong psychometric properties, including high internal consistency and good construct validity. It reliably measures self-compassion across six variables: self-kindness, self-judgment, common humanity, isolation, mindfulness, and over-identification.

The OAS [52,53] and ESS [54] were both administered to assess participants' experiences of shame, including feelings of worthlessness and self-criticism [28]. These are two distinct psychological assessment tools used to explore the complex emotion of shame, but they differ in their primary focus and purpose. While the OAS assesses the external social aspect of shame, the ESS delves into the internal emotional landscape of the individual, making them both valuable tools for understanding shame from different angles in psychological research and clinical applications. Additionally, demographic information including leisure and exercise habits, was collected from the participants.

Statistical analysis

Descriptive statistics were computed to summarize demographic characteristics and scores on the study variables. Independent samples t-test was conducted to examine potential differences in mental well-being and shame scores across various family characteristics and leisure activities categories. This exploratory analysis provided an initial understanding of the associations between these factors and shame experiences within the sample. Subsequently, a path analysis was employed to examine the relationships between demographic variables (age, gender), oral health indicators (GOHAI subscales), psychological resilience, self-compassion, and shame (ESS and OAS).

In this context, self-compassion and resilience were regressed on the three GOHAI subscales, while the GOHAI subscales, along with self-compassion and resilience, were regressed on shame. Age and gender were included as control variables across all paths to account for their potential confounding effects. Path coefficients, standard errors, and significance levels were estimated to assess the direct and indirect effects of predictor variables on outcome variables. The regression coefficients of the completely standardized solution were also computed and presented, enabling comparisons between the effects of different independent variables on the same dependent variable.

All data were analyzed using the R statistical language [55,56] equipped with the lavaan package [57]. The path analysis model was optimized using the maximum likelihood estimator and the NLMINB optimization method, selected by default by lavaan for its efficiency and accuracy in estimating parameters within complex linear models.

## Results

Resilience, self-compassion, and shame: exploring family dynamics and leisure activities

Table 1 presents the differences in resilience, self-compassion, and shame among elderly individuals based on various characteristics. Respondents who perceive old age as difficult tended to have lower resilience (p = 0.061) and significantly lower self-compassion (p = 0.009) compared to those who do not perceive old age as difficult. Furthermore, the respondents with siblings showed higher self-compassion compared to those without (p = 0.011), while having a daughter or a daughter living nearby was related to higher resilience (p = 0.170 and p = 0.019 respectively).

**Table 1:** Resilience, self-compassion, and shame across various family characteristics and leisure activities ^(1)^Independent samples t-test

Questionnaire item (yes/no)		Resilience			Self-compassion			Experiential (internal) shame			Other as shamer (social)		
		Yes	No	p^(1)^	Yes	No	p^(1)^	Yes	No	p^(1)^	Yes	No	p^(1)^
Old age is difficult (186/18)		60 (19.3)	68.9 (17.4)	0.061	3.13 (0.39)	3.38 (0.41)	0.009	50.4 (16.6)	48.8 (16.2)	0.702	18.1 (13.1)	20.1 (11.9)	0.529
Family													
	Siblings (184/18)	61.3 (18.0)	53.0 (28.1)	0.234	3.17 (0.4)	2.93 (0.42)	0.011	50.0 (16.2)	55.2 (19.3)	0.197	18.0 (12.2)	22.6 (19.5)	0.329
	Children (190/14)	61.3 (18.9)	54.1 (23.3)	0.182	3.16 (0.4)	2.97 (0.40)	0.082	49.8 (16.1)	55.6 (22.3)	0.211	17.8 (12.3)	25.8 (19.1)	0.141
	Have a daughter (148/56)	61.9 (18.5)	57.8 (21.0)	0.170	3.15 (0.38)	3.14 (0.40)	0.787	50.4 (16.5)	49.8 (17.0)	0.836	17.9 (12.4)	19.5 (14.6)	0.390
	Daughter lives near (61/143)	65.6 (17.4)	58.7 (19.7)	0.019	3.17 (0.4)	3.14 (0.40)	0.577	51.0 (16.6)	49.9 (16.6)	0.645	16.9 (8.79)	18.9 (14.4)	0.241
	Have a son (130/73)	60.9 (18.3)	60.1 (20.8)	0.782	3.16 (0.4)	3.14 (0.41)	0.753	49.6 (15.9)	51.5 (17.6)	0.431	18.7 (13.5)	17.8 (12.1)	0.654
	Son lives near (63/138)	62.8 (18.1)	59.4 (19.6)	0.241	3.20 (0.4)	3.13 (0.40)	0.259	48.4 (14.2)	51.4 (17.5)	0.239	19.6 (13.7)	17.8 (12.5)	0.331
Leisure and exercise													
	Holidays (82/121)	64.2 (18.6)	58.2 (19.2)	0.028	3.17 (0.4)	3.14 (0.40)	0.503	51.9 (18.2)	49.3 (15.2)	0.275	16.0 (13.5)	20.0 (12.4)	0.036
	Walking (137/67)	62.8 (18.8)	56.7 (19.7)	0.036	3.19 (0.4)	3.06 (0.35)	0.030	47.7 (15.7)	55.4 (17.2)	0.002	17.4 (13.0)	20.2 (13.0)	0.176
	Garden (136/68)	60.9 (20.0)	60.6 (17.9)	0.941	3.14 (0.4)	3.17 (0.38)	0.611	49.0 (16.2)	52.6 (17.2)	0.143	18.3 (13.4)	18.3 (12.2)	0.905
	Friends in the past (181/23)	62.9 (17.2)	43.8 (25.4)	0.002	3.17 (0.4)	2.99 (0.34)	0.046	49.7 (16.7)	54.4 (15.4)	0.195	18.0 (12.4)	20.8 (17.2)	0.443
	Friends today (155/49)	64.9 (16.2)	47.9 (22.5)	<0.001	3.21 (0.4)	2.96 (0.37)	<0.001	49.7 (17.0)	51.7 (15.3)	0.474	17.2 (12.3)	21.8 (14.7)	0.029

Regarding family characteristics, there were no significant differences in shame scores between individuals who perceived old age as difficult and those who did not, nor were there significant differences based on having siblings, children, daughters, sons, or proximity of daughters or sons. However, for leisure and exercise activities, some notable differences emerged. Specifically, individuals who engaged in walking (p = 0.002) or went on holidays (p = 0.036) exhibited significantly lower levels of internal shame. Similarly, individuals who reported having friends today experienced significantly lower levels of social shame compared to those who did not (p = 0.029), suggesting a potential association between social interactions and shame experiences. Overall, a pattern emerges wherein individuals who engage in more social and leisure activities demonstrate better mental well-being, as evidenced by higher levels of resilience and self-compassion and lower levels of shame than those who do not.

Path analysis: mental well-being's effect on oral health and shame feelings

The mean score, the internal consistency, and the Pearson correlation coefficient of the model variables are presented in Table 2. All psychometric scales exhibited acceptable to excellent internal reliability. The maximum likelihood procedure ended normally after 131 iterations. The results of the path model are presented in Table 3.

**Table 2:** Descriptive statistics of path model variables ^*^p<0.05 (statistically significant at the 5% level); ^**^p<0.01 (statistically significant at the 1% level)

		Reliability	Gender		Total (N = 200)	Psychological well-being		Self-perceived oral health			Shame		
						CDRISC	SCS	PF	PS	PD	ESS	OAS	
			Female (N = 121)	Male (N = 79)									
Psychological well-being													
	Resilience (CDRISC)	0.950	60.2 (20.1)	62.1 (18.3)	60.9 (19.3)								
	Self-compassion (SCS)	0.890	3.1 (0.39)	3.2 (0.40)	3.2 (0.40)	0.561^**^							
Self-perceived oral health													
	Physical function (PF)	0.760	12.2 (4.6)	12.7 (4.3)	12.4 (4.4)	0.324^**^	0.273^**^						
	Psychosocial function (PS)	0.738	15.1 (5.5)	14.8 (5.5)	15.0 (5.5)	0.333^**^	0.329^**^	0.642^**^					
	Pain/discomfort (PD)	0.568	10.0 (3.0)	9.7 (3.3)	9.9 (3.1)	0.183^**^	0.146^*^	0.669^**^	0.635^**^				
Shame													
	Experiential shame (ESS)	0.950	52.0 (16.8)	47.5 (16.1)	50.2 (16.6)	-0.183^**^	-0.311^**^	-0.203^**^	-0.300^**^	-0.220^**^			
	Other as shamer (OAS)	0.940	18.6 (13.0)	17.9 (13.3)	18.3 (13.1)	-0.314^**^	-0.320^**^	-0.208^**^	-0.193^**^	-0.172^*^	0.432^**^		
Age		-	74.3 (7.2)	74 (7.0)	74.2 (7.1)	-0.172^*^	-0.169^*^	-0.368^**^	-0.252^**^	-0.210^**^	-0.011	0.002	

**Table 3:** Path analysis results ^(*)^Completely standardized solution

		b	SE	z	p	95% CI		std^(*)^	R^2^
						Lower	Upper		
Self-compassion									0.033
	Age	-0.009	0.004	-2.417	0.016	-0.017	-0.002	-0.168	
	Gender	0.054	0.056	0.969	0.332	-0.055	0.164	0.067	
Resilience									0.032
	Age	-0.469	0.190	-2.464	0.014	-0.843	-0.096	-0.171	
	Gender	1.839	2.747	0.670	0.503	-3.545	7.223	0.047	
Physical function									0.213
	Age	-0.197	0.040	-4.914	0.000	-0.276	-0.119	-0.314	
	Gender	0.333	0.569	0.585	0.558	-0.783	1.449	0.037	
	Self-compassion	1.098	0.858	1.279	0.201	-0.584	2.780	0.097	
	Resilience	0.049	0.017	2.802	0.005	0.015	0.083	0.213	
Psychosocial function									0.176
	Age	-0.145	0.051	-2.855	0.004	-0.245	-0.046	-0.187	
	Gender	-0.592	0.723	-0.819	0.413	-2.009	0.825	-0.053	
	Self-compassion	2.672	1.090	2.452	0.014	0.536	4.808	0.191	
	Resilience	0.056	0.022	2.516	0.012	0.012	0.099	0.196	
Pain/discomfort									0.071
	Age	-0.080	0.031	-2.602	0.009	-0.139	-0.020	-0.181	
	Gender	-0.395	0.434	-0.912	0.362	-1.245	0.454	-0.062	
	Self-compassion	0.375	0.654	0.574	0.566	-0.906	1.656	0.047	
	Resilience	0.021	0.013	1.552	0.121	-0.005	0.047	0.128	
Internal shame									0.174
	Physical function	0.102	0.372	0.273	0.785	-0.628	0.832	0.027	
	Psychosocial function	-0.628	0.282	-2.228	0.026	-1.180	-0.075	-0.208	
	Pain/discomfort	-0.558	0.504	-1.108	0.268	-1.546	0.429	-0.104	
	Age	-0.273	0.163	-1.672	0.094	-0.593	0.047	-0.116	
	Gender	-4.434	2.207	-2.009	0.045	-8.760	-0.109	-0.131	
	Self-compassion	-11.060	3.356	-3.295	0.001	-17.639	-4.482	-0.262	
	Resilience	0.026	0.069	0.375	0.708	-0.109	0.160	0.030	
External (social) shame									0.152
	Physical function	-0.264	0.297	-0.889	0.374	-0.847	0.318	-0.090	
	Psychosocial function	0.039	0.225	0.173	0.863	-0.402	0.479	0.016	
	Pain/discomfort	-0.348	0.402	-0.865	0.387	-1.136	0.440	-0.083	
	Age	-0.203	0.130	-1.558	0.119	-0.459	0.052	-0.110	
	Gender	-0.078	1.761	-0.044	0.965	-3.530	3.375	-0.003	
	Self-compassion	-6.907	2.678	-2.579	0.010	-12.157	-1.658	-0.208	
	Resilience	-0.120	0.055	-2.190	0.029	-0.227	-0.013	-0.177	

Age had a significant negative association with self-compassion (b = -0.009, b_std_ = -0.168, p = 0.016) and resilience (b = -0.469, b_std_ = -0.171, p = 0.014), indicating that older individuals tended to have lower levels of psychological well-being. In contrast, no significant association was found between gender and these psychological characteristics (p>0.05).

Concerning the three GOHAI oral health subscales, significant associations were found with age, self-compassion, and resilience. Specifically, older age was negatively associated with physical function (b = -0.197, b_std_ = -0.314, p<0.001), psychosocial function (b = -0.145, b_std_ = -0.187, p = 0.004), and pain/discomfort (b = -0.080, b_std_ = -0.181, p = 0.009), indicating that older individuals reported poorer oral health in all three aspects. On the other hand, higher levels of self-compassion were positively associated with psychosocial function (b = 2.672, b_std_ = 0.191, p = 0.014). Resilience showed a significant positive association with physical function (b = 0.049, b_std_ = 0.213, p = 0.005) and psychosocial function (b = 0.056, b_std_ = 0.196, p = 0.012), indicating that higher levels of an overall better psychological well-being are associated with better physical and psychosocial function related to oral health.

Concerning shame, significant associations were found with age, gender, self-compassion, and resilience. Specifically, higher levels of self-compassion were negatively associated with internal shame (b = -11.060, b_std_ = -0.262, p = 0.001), and external (social) shame (b = -6.907, b_std_ = -0.208, p = 0.010) suggesting that individuals with higher self-compassion scores reported lower experiences of shame. No significant association was found between age and internal shame or external (social) shame (p>0.05). Additionally, resilience showed a significant negative association with external (social) shame (b = -0.120, b_std_ = -0.177, p = 0.029), suggesting that higher levels of resilience were associated with lower tendencies to shame others. A gender effect was also reported regarding internal shame, where males were characterized by lower internal shame than females (b = -4.434, b_std_ = -0.131, p = 0.045).

## Discussion

Our study contributes to the existing literature by corroborating previous findings indicating that advancing age is associated with declining physical function and psychosocial well-being [58]. Additionally, our results align with prior research demonstrating a negative correlation between older age and levels of psychological well-being [59,60]. As evidenced by prior research [61,62], people with elevated levels of resilience and self-compassion show enhanced abilities to maintain positive attitudes and behaviors toward oral care, engage in proactive health-seeking actions, and adeptly manage oral health challenges. A noteworthy discovery in our study is the substantial positive impact of resilience and self-compassion on both physical and psychosocial dimensions of oral health-related quality of life, effectively counteracting the adverse effects of aging. Consequently, even as individuals age and confront escalating oral health difficulties, those with resilience and self-compassion may perceive themselves as better equipped to navigate their oral health needs and maintain a sense of agency and control over their circumstances.

Moreover, our findings align with existing literature [63-66], suggesting that engagement in social and leisure activities can significantly enhance overall mental well-being. From the perspective of our study, it is anticipated that such activities may exert a corresponding positive influence on the physical and psychosocial aspects of oral health, as well as on feelings of shame. These insights underscore the potential benefits of promoting resilience, self-compassion, and social and leisure activities as integral components of interventions to enhance oral health and well-being across the lifespan.

Our study further supports the influence of psychological well-being on oral health-related quality of life, with higher levels of self-compassion and resilience linked to favorable outcomes across both physical and psychosocial dimensions of the GOHAI scale [67,68]. Also, our findings corroborate the well-documented association between psychological well-being and experiences of shame [69-71]. Specifically, elevated levels of self-compassion and resilience were associated with reduced levels of shame, endorsing the hypothesis that these traits may serve as effective emotion regulation strategies, mitigating negative emotions such as shame [72-76]. Our findings further suggest that resilience, by fostering a sense of internal strength and resourcefulness, may act as a buffer against the adverse impact of shame, empowering individuals to navigate social interactions with greater confidence [77]. Similarly, cultivating self-kindness, common humanity, and mindfulness, characteristic of self-compassionate individuals, may reduce susceptibility to internalizing shame and enhance resilience in the face of social challenges [78-80].

Of note, oral health subscales did not emerge as significant predictors of external (social) shame. This underscores the role of resilience and self-compassion in elucidating the connections between oral health and shame, emphasizing the importance of addressing psychological well-being in interventions aimed at reducing shame and improving social functioning among older individuals. That is, while oral health undoubtedly shapes individuals' experiences of shame, particularly in social settings where visible oral health issues may elicit stigma or embarrassment, our findings underscore the key role of psychological resources such as resilience and self-compassion in shaping individuals' responses to shame-inducing situations. These findings align with previous research highlighting the positive impact of psychological resources on health outcomes [13,81,82], emphasizing the importance of holistic approaches addressing both physical and psychological aspects of well-being in promoting overall health and resilience among older populations. Even more, these results are further supported by the finding that individuals who engage in more social and leisure activities exhibit better mental well-being, as evidenced by higher levels of resilience and self-compassion.

In the same vein, the finding that self-compassion outweighs the psychosocial function subscale in predicting internal shame suggests that the way individuals relate to themselves and their internal experiences may have a more profound impact on their emotional well-being than their perceived satisfaction with social interactions or psychological distress related to oral health issues. Individuals who cultivate self-compassion are better equipped to cope with the emotional challenges associated with oral health problems, fostering greater emotional resilience and psychological flourishing [83,84], reinforcing the idea that self-compassion contributes to better coping strategies and resilience in the face of adversity [85], and emphasizing the role of self-compassion in maintaining mental and physical health, particularly in managing chronic health problems [86]. This resilience and self-kindness enable individuals to navigate social interactions and maintain positive relationships despite oral health-related limitations, enhancing psychosocial functioning and subjective well-being, even in the presence of age-related declines in oral health.

The main contributions of the study are outlined below:

(1) Self-compassion's impact on emotional well-being: it highlights that self-compassion has a stronger influence on reducing internal shame compared to perceived satisfaction with social interactions or psychological distress related to oral health issues.

(2) Coping with emotional challenges: it emphasizes that individuals who cultivate self-compassion are better equipped to handle the emotional difficulties associated with oral health problems, leading to greater emotional resilience and psychological flourishing.

(3) Navigating social interactions: this study suggests that self-compassion fosters resilience and self-kindness, enabling individuals to maintain positive social relationships and interactions despite limitations caused by oral health issues.

(4) Enhanced psychosocial functioning and well-being: it concludes that self-compassion contributes to improved psychosocial functioning and subjective well-being, even in the setting of age-related declines in oral health.

Recommendations

The importance of addressing psychological well-being as an integral component of geriatric care to enhance the overall quality of life and well-being in elder individuals is emphasized. Specifically, interventions aimed at enhancing resilience and self-compassion may hold promise for mitigating the negative impact of age-related oral health issues and promoting adaptive coping strategies among elderly populations. Suggested interventions aimed at enhancing resilience and self-compassion in elderly populations to mitigate the negative impact of age-related oral health issues and promote adaptive coping strategies include:

(1) Mindfulness-based stress reduction: it can increase self-awareness, reduce stress, and improve emotional regulation. This, in turn, can enhance self-compassion and resilience, helping older adults better manage the emotional challenges of oral health issues.

(2) Compassion-focused therapy: by fostering self-kindness and reducing self-criticism, compassion-focused therapy can improve emotional well-being and resilience, enabling older adults to better cope with the psychological and social impacts of oral health problems.

(3) Resilience training programs: resilience training can empower older adults with skills to bounce back from setbacks and maintain a positive outlook despite oral health challenges, thereby enhancing their coping strategies.

(4) Psychoeducation on oral health and self-compassion: increased knowledge can empower individuals to take proactive steps in managing their oral health and emotional well-being, leading to improved coping strategies and resilience.

These interventions, by fostering self-compassion and resilience, can help older adults better cope with the emotional and social challenges of age-related oral health issues, ultimately leading to improved psychosocial functioning and well-being. Further research is warranted to explore the mechanisms underlying these associations and to develop targeted interventions tailored to the unique needs of aging populations.

Limitations

Several limitations of this study should be acknowledged. Firstly, the cross-sectional nature of the data precludes the establishment of causality or the assessment of temporal relationships between variables. Longitudinal studies are needed to elucidate the directionality of the observed associations and to capture changes in psychological well-being and oral health outcomes over time. Furthermore, the study sample consisted of elder individuals from a specific geographic region, which may limit the generalizability of the findings to other populations or cultural contexts. Finally, while our model accounted for several key demographic and psychological factors, there may be additional variables or pathways that influence oral health outcomes not captured in our analysis. Future research could explore other potential predictors or moderators of oral health-related quality of life to provide a more nuanced understanding of these relationships.

## Conclusions

Resilience and self-compassion emerged as significant predictors of oral health-related quality of life and experiences of shame, suggesting that psychological resources play a critical role in shaping individuals' perceptions of and responses to oral health challenges. These findings underscore the multifaceted nature of oral health-related quality of life and its associations with various psychosocial factors, highlighting the importance of considering both intrapersonal and interpersonal dimensions of well-being in the context of oral health care for aging populations.

## References

[1] Mitchell E, Walker R (2020). Global ageing: successes, challenges and opportunities. Br J Hosp Med (Lond).

[2] Brown L, Huffman JC, Bryant C (2019). Self-compassionate aging: a systematic review. Gerontologist.

[3] Cha JE, Boggiss AL, Serlachius AS, Cavadino A, Kirby JN, Consedine NS (2022). A systematic review on mediation studies of self-compassion and physical health outcomes in non-clinical adult populations. Mindfulness.

[4] Homan KJ, Sirois FM (2017). Self-compassion and physical health: exploring the roles of perceived stress and health-promoting behaviors. Health Psychol Open.

[5] Misurya I, Misurya P, Dutta A (2020). The effect of self-compassion on psychosocial and clinical outcomes in patients with medical conditions: a systematic review. Cureus.

[6] Neff KD (2003). The development and validation of a scale to measure self-compassion. Self Identity.

[7] Sirois FM (2020). The association between self-compassion and self-rated health in 26 samples. BMC Public Health.

[8] Windle G (2011). What is resilience? A review and concept analysis. Rev Clin Gerontol.

[9] Bonanno GA (2004). Loss, trauma, and human resilience: have we underestimated the human capacity to thrive after extremely aversive events?. Am Psychol.

[10] Southwick SM, Bonanno GA, Masten AS, Panter-Brick C, Yehuda R (2014). Resilience definitions, theory, and challenges: interdisciplinary perspectives. Eur J Psychotraumatol.

[11] Breines JG, Chen S (2012). Self-compassion increases self-improvement motivation. Pers Soc Psychol Bull.

[12] Neff K: Self-Compassion (2003). Self-compassion: an alternative conceptualization of a healthy attitude toward oneself. Self Identity.

[13] Allen AB, Goldwasser ER, Leary MR (2012). Self-compassion and well-being among older adults. Self Identity.

[14] Cunha M, Parente L, Galhardo A, Couto M (2017). Self-compassion, well-being and health in elderly: are there related?. Eur Psychiatry.

[15] Brinkhof LP, Chambon M, Ridderinkhof KR, Van Harreveld F, Murre JMJ, Krugers HJ, De Wit S (2023). Resilience among older individuals in the face of adversity: how demographic and trait factors affect mental-health constructs and their temporal dynamics. Clin Psychol Sci.

[16] MacLeod S, Musich S, Hawkins K, Alsgaard K, Wicker ER (2016). The impact of resilience among older adults. Geriatr Nurs.

[17] Merchant RA, Aprahamian I, Woo J, Vellas B, Morley JE (2022). Editorial: resilience and successful aging. J Nutr Health Aging.

[18] Hodgetts J, McLaren S, Bice B, Trezise A (2021). The relationships between self-compassion, rumination, and depressive symptoms among older adults: the moderating role of gender. Aging Ment Health.

[19] Karakasidou E, Raftopoulou G, Stalikas A (2020). Investigating differences in self-compassion levels: effects of gender and age in a Greek adult sample. Psychol J Hell Psychol Soc.

[20] Sardella A, Lenzo V, Basile G, Musetti A, Franceschini C, Quattropani MC (2022). Gender and psychosocial differences in psychological resilience among a community of older adults during the COVID-19 pandemic. J Pers Med.

[21] Baiju RM, Peter E, Varghese NO, Sivaram R (2017). Oral health and quality of life: current concepts. J Clin Diagn Res.

[22] Christensen K, Doblhammer G, Rau R, Vaupel JW (2009). Ageing populations: the challenges ahead. Lancet.

[23] Kossioni AE (2018). The association of poor oral health parameters with malnutrition in older adults: a review considering the potential implications for cognitive impairment. Nutrients.

[24] Peres MA, Macpherson LM, Weyant RJ (2019). Oral diseases: a global public health challenge. Lancet.

[25] Sander M, Oxlund B, Jespersen A, Krasnik A, Mortensen EL, Westendorp RG, Rasmussen LJ (2015). The challenges of human population ageing. Age Ageing.

[26] Doughty J, Macdonald ME, Muirhead V, Freeman R (2023). Oral health-related stigma: describing and defining a ubiquitous phenomenon. Community Dent Oral Epidemiol.

[27] Guimarães MO, Drumond CL, Nunes LS, Oliveira ES, Zarzar PM, Ramos-Jorge ML, Vieira-Andrade RG (2021). Prevalence of oral health-related shame and associated factors among Brazilian schoolchildren. Braz Oral Res.

[28] Kim S, Thibodeau R, Jorgensen RS (2011). Shame, guilt, and depressive symptoms: a meta-analytic review. Psychol Bull.

[29] Alexander B, Brewin CR, Vearnals S, Wolff G, Leff J (1999). An investigation of shame and guilt in a depressed sample. Br J Med Psychol.

[30] Cheung MSP, Gilbert P, Irons C (2004). An exploration of shame, social rank and rumination in relation to depression. Personal Individ Differ.

[31] Matos M, Pinto-Gouveia J (2010). Shame as a traumatic memory. Clin Psychol Psychother.

[32] Tangney JP, Wagner P, Gramzow R (1992). Proneness to shame, proneness to guilt, and psychopathology. J Abnorm Psychol.

[33] Matos M, Pinto-Gouveia J, Gilbert P (2013). The effect of shame and shame memories on paranoid ideation and social anxiety. Clin Psychol Psychother.

[34] Harman R, Lee D (2010). The role of shame and self-critical thinking in the development and maintenance of current threat in post-traumatic stress disorder. Clin Psychol Psychother.

[35] Skårderud F (2007). Shame and pride in anorexia nervosa: a qualitative descriptive study. Eur Eat Disord Rev.

[36] Troop NA, Allan S, Serpell L, Treasure JL (2008). Shame in women with a history of eating disorders. Eur Eat Disord Rev.

[37] Rüsch N, Lieb K, Göttler I (2007). Shame and implicit self-concept in women with borderline personality disorder. Am J Psychiatry.

[38] Berggren U, Meynert G (1984). Dental fear and avoidance: causes, symptoms, and consequences. J Am Dent Assoc.

[39] Kandelman D, Petersen PE, Ueda H (2008). Oral health, general health, and quality of life in older people. Spec Care Dentist.

[40] Rouxel P, Heilmann A, Demakakos P, Aida J, Tsakos G, Watt RG (2017). Oral health-related quality of life and loneliness among older adults. Eur J Ageing.

[41] Smith JM, Sheiham A (1979). How dental conditions handicap the elderly. Community Dent Oral Epidemiol.

[42] Martins AB, Dos Santos CM, Hilgert JB, de Marchi RJ, Hugo FN, Pereira Padilha DM (2011). Resilience and self-perceived oral health: a hierarchical approach. J Am Geriatr Soc.

[43] Teixeira MF, Martins AB, Celeste RK, Hugo FN, Hilgert JB (2015). Association between resilience and quality of life related to oral health in the elderly. Rev Bras Epidemiol.

[44] Tsironis C, Mantzoukas S, Tatsis F, Kourakos M, Diamantopoulos E, Dragioti E, Gouva M (2024). Exploring the mediating role of shame in the link between oral health and psychopathology in older adults. Int J Psychiatr.

[45] Guarnizo-Herreño CC, Watt RG, Pikhart H, Sheiham A, Tsakos G (2013). Socioeconomic inequalities in oral health in different European welfare state regimes. J Epidemiol Community Health.

[46] Chiu CJ, Hu JC, Lo YH, Chang EY (2020). Health promotion and disease prevention interventions for the elderly: a scoping review from 2015-2019. Int J Environ Res Public Health.

[47] Dickens AP, Richards SH, Greaves CJ, Campbell JL (2011). Interventions targeting social isolation in older people: a systematic review. BMC Public Health.

[48] Liljas AE, Walters K, Jovicic A, Iliffe S, Manthorpe J, Goodman C, Kharicha K (2017). Strategies to improve engagement of 'hard to reach' older people in research on health promotion: a systematic review. BMC Public Health.

[49] Tkatch R, Musich S, MacLeod S, Alsgaard K, Hawkins K, Yeh CS (2016). Population health management for older adults: review of interventions for promoting successful aging across the health continuum. Gerontol Geriatr Med.

[50] Tubert-Jeannin S, Riordan PJ, Morel-Papernot A, Porcheray S, Saby-Collet S (2003). Validation of an oral health quality of life index (GOHAI) in France. Community Dent Oral Epidemiol.

[51] Atchison K, Dolan T (1990). Development of the Geriatric Oral Health Assessment Index. J Dent Educ.

[52] Allan S, Gilbert P, Goss K (1994). An exploration of shame measures—II: psychopathology. Personal Individ Differ.

[53] Goss K, Gilbert P, Allan S (1994). An exploration of shame measures—I: the other as Shamer scale. Personal Individ Differ.

[54] Andrews B, Qian M, Valentine JD (2015). Experience of Shame Scale.

[55] (2024). Rstudio: integrated development environment for R. Posit Softw PBC Boston MA. Published Online First.

[56] (2024). A language and environment for statistical computing. R Foundation for Statistical Computing, Vienna. https://www.r-project.org/.

[57] Rosseel Y (2012). lavaan: an R package for structural equation modeling. J Stat Softw.

[58] Janto M, Iurcov R, Daina CM (2022). Oral health among elderly, impact on life quality, access of elderly patients to oral health services and methods to improve oral health: a narrative review. J Pers Med.

[59] Charles ST, Rush J, Piazza JR, Cerino ES, Mogle J, Almeida DM (2023). Growing old and being old: emotional well-being across adulthood. J Pers Soc Psychol.

[60] Kang H, Kim H (2022). Ageism and psychological well-being among older adults: a systematic review. Gerontol Geriatr Med.

[61] Ferrari M, Dal Cin M, Steele M (2017). Self-compassion is associated with optimum self-care behaviour, medical outcomes and psychological well-being in a cross-sectional sample of adults with diabetes. Diabet Med.

[62] Sotiropoulou K, Patitsa C, Giannakouli V, Galanakis M, Koundourou C, Tsitsas G (2023). Self-compassion as a key factor of subjective happiness and psychological well-being among Greek adults during COVID-19 lockdowns. Int J Environ Res Public Health.

[63] Cummings SM (2002). Predictors of psychological well-being among assisted-living residents. Health Soc Work.

[64] Fredrickson BL (2001). The role of positive emotions in positive psychology: the broaden-and-build theory of positive emotions. Am Psychol.

[65] Iwasaki Y (2006). Counteracting stress through leisure coping: a prospective health study. Psychol Health Med.

[66] Takiguchi Y, Matsui M, Kikutani M, Ebina K (2023). The relationship between leisure activities and mental health: the impact of resilience and COVID-19. Appl Psychol Health Well Being.

[67] Goh V, Hassan FW, Baharin B, Rosli TI (2022). Impact of psychological states on periodontitis severity and oral health-related quality of life. J Oral Sci.

[68] Ou-Yang ZY, Feng Y, Yang YF (2023). Oral health-related quality of life among Chinese chronic orofacial pain patients with psychological health problems: a moderated mediation model. Int J Environ Res Public Health.

[69] Röthlin P, Horvath S, Messerli T, Krieger T, Berger T, Birrer D (2023). Associations of self-compassion with shame, guilt, and training motivation after sport-specific daily stress - a smartphone study. Int J Sport Exerc Psychol.

[70] Sedighimornani N, Rimes KA, Verplanken B (2019). Exploring the relationships between mindfulness, self-compassion, and shame. SAGE Open Med.

[71] Van Vliet KJ (2008). Shame and resilience in adulthood: a grounded theory study. J Couns Psychol.

[72] Gilbert P (2000). The relationship of shame, social anxiety and depression: the role of the evaluation of social rank. Clin Psychol Psychother.

[73] Kelly AC, Zuroff DC, Shapira LB (2009). Soothing oneself and resisting self-attacks: the treatment of two intrapersonal deficits in depression vulnerability. Cogn Ther Res.

[74] Mosewich AD, Kowalski KC, Sabiston CM, Sedgwick WA, Tracy JL (2011). Self-compassion: a potential resource for young women athletes. J Sport Exerc Psychol.

[75] Proeve M, Anton R, Kenny M (2018). Effects of mindfulness-based cognitive therapy on shame, self-compassion and psychological distress in anxious and depressed patients: a pilot study. Psychol Psychother.

[76] Woods H, Proeve M (2014). Relationships of mindfulness, self-compassion, and meditation experience with shame-proneness. J Cogn Psychother.

[77] Johnson J, Panagioti M, Bass J, Ramsey L, Harrison R (2017). Resilience to emotional distress in response to failure, error or mistakes: a systematic review. Clin Psychol Rev.

[78] Ferreira C, Matos M, Duarte C, Pinto-Gouveia J (2014). Shame memories and eating psychopathology: the buffering effect of self-compassion. Eur Eat Disord Rev.

[79] Johnson EA, O’Brien KA (2013). Self-compassion soothes the savage ego-threat system: effects on negative affect, shame, rumination, and depressive symptoms. J Soc Clin Psychol.

[80] Zhang H, Carr ER, Garcia-Williams AG (2018). Shame and depressive symptoms: self-compassion and contingent self-worth as mediators?. J Clin Psychol Med Settings.

[81] Pellerin N, Raufaste E (2020). Psychological resources protect well-being during the COVID-19 pandemic: a longitudinal study during the French lockdown. Front Psychol.

[82] Phillips WJ, Hine DW (2021). Self-compassion, physical health, and health behaviour: a meta-analysis. Health Psychol Rev.

[83] Allen AB, Leary MR (2010). Self-compassion, stress, and coping. Soc Personal Psychol Compass.

[84] Neff KD (2011). Self‐compassion, self‐esteem, and well‐being. Soc Personal Psychol Compass.

[85] Terry ML, Leary MR, Mehta S, Henderson K (2013). Self-compassionate reactions to health threats. Pers Soc Psychol Bull.

[86] Sirois FM, Hirsch JK (2019). Self-compassion and adherence in five medical samples: the role of stress. Mindfulness (N Y).

